# Genome sequencing reveals the prevalence of Fabry disease–causing variants in Japan: Evidence from the Tohoku Medical Megabank

**DOI:** 10.1016/j.gimo.2026.104490

**Published:** 2026-08-07

**Authors:** Kai Susukita, Kotaro Nochioka, Masato Takase, Saori Yamamoto, Kazuki Kumada, Hideaki Suzuki, Kinuko Ohneda, Kenichi Noguchi, Yoko Izumi, Soichi Ogishima, Naoki Nakaya, Yoshikatsu Eto, Atsushi Hozawa, Satoshi Yasuda

**Affiliations:** 1 Department of Cardiovascular Medicine, Tohoku University Graduate School of Medicine, Sendai, Japan; 2 Tohoku Medical Megabank Organization, Tohoku University, Sendai, Japan; 3 Advanced Clinical Research Center and Asian LSD Center, Institute of Neurological disorders, Fukushima, Japan

**Keywords:** Fabry disease, Genome sequencing, Globotriaosylsphingosine, Prevalence

## Abstract

**Background:**

Fabry disease (FD) is an X-linked lysosomal storage disorder caused by deficient α-galactosidase A activity and characterized by multisystem involvement. Although considered rare, with reported prevalence ranging from 1 in 40,000 to 1 in 140,000, its true frequency in the general population remains uncertain. We aimed to determine the prevalence of FD-causing variants in a community-based cohort from the Tohoku Medical Megabank, Japan.

**Methods:**

Genome sequencing was performed in 68,615 individuals. Variant pathogenicity was classified according to American College of Medical Genetics and Genomics/Association for Molecular Pathology guidelines. Baseline demographics, serum α-galactosidase A (GLA) activity, plasma globotriaosylsphingosine, and self-reported health data were assessed.

**Results:**

Pathogenic GLA variants were identified in 4 individuals and a likely pathogenic variant in 1 individual (mean age 49.3 ± 15.9 years); all were female, corresponding to a prevalence of 1 in 13,723. All pathogenic variants were c.902G>A, associated with the cardiac phenotype, whereas the likely pathogenic variant was c.1076T>C. Mean plasma GLA activity was 5.64 ± 1.73 mmol/hr/mL in pathogenic variant carriers and 8.36 mmol/hr/mL in the likely pathogenic carrier. Plasma globotriaosylsphingosine levels were 3.7 ± 0.79 ng/mL and 0.96 ng/mL, respectively.

**Conclusion:**

Population based genome sequencing data suggest a higher prevalence of FD-causing variants in Japan than previously reported, highlighting potential underdiagnosis of late-onset FD in women.

## Introduction

Fabry disease (FD) is a rare X-linked lysosomal storage disorder caused by pathogenic variants in the *GLA* gene, resulting in impaired activity of α-galactosidase A (GLA).^1^ This enzymatic deficiency leads to the progressive accumulation of globotriaosylceramide and related metabolites in vascular endothelial cells and multiple organs, including the heart, kidneys, and nervous system.^1^^,^^2^ The prevalence of FD in the general population has traditionally been estimated to be approximately 1 in 40,000 to 140,000 individuals.^1^^,^^3^ Although newborn screening programs, particularly in Taiwan, have provided well-defined estimates of Fabry disease prevalence and have revealed a substantially higher frequency of late-onset variants, population-based genomic data remain limited in other Asian populations.^1^^,^^4^ This study aimed to determine the prevalence of FD–causing variants in genome sequencing data from a community-based cohort in the Tohoku Medical Megabank, Japan.

## Methods

The Tohoku Medical Megabank (TMM) Project aims to integrate population genomics, medical genetics, and prospective cohort studies to establish a critical infrastructure for the advancement of precision medicine. A key strength of the TMM Project is its linkage of genomic data with detailed health questionnaires, biospecimens, and longitudinal clinical information in a large community-based population. The TMM Community Cohort Study is a general population-based prospective cohort comprising individuals aged ≥20 years from Miyagi Prefecture in northeastern Japan. The design, methods, and other details of this study have been described elsewhere.^5^^,^^6^ Briefly, initiated in May 2013, the study enrolled more than 50,000 participants through March 2016. The type 1 survey was conducted mainly at municipal, specific health check-up sites (*n* = 40,433), with additional survey sessions held on separate dates to accommodate overflow at venue sites (*n* = 664). In contrast, the type 2 survey was conducted at designated Community Support Centers (*n* = 13,855), where participants voluntarily underwent more detailed physiological and biochemical examinations and biological specimen collection.^5^^,^^6^ Participants provided lifestyle and other health-related information through self-administered questionnaires and submitted blood and urine samples.

We used the quality-controlled, jointly genotyped genome sequencing multisample variant call format call set provided by the TMM Project (69KJPN), aligned to the GRCh38 reference genome build.^7^ This publicly available resource comprises large-scale population-based genomic data generated under a standardized sequencing and quality-control framework, enabling genotype-first assessment of rare disease–associated variants in the general population. Variant calling and quality control, including read alignment, joint genotyping, and variant-level filtering, were performed by TMM using a Genome Analysis Toolkit Best Practices–based pipeline as described previously.^7^, ^8^, ^9^ We did not perform realignment or variant recalling from raw sequencing reads (FASTQ/BAM); all downstream analyses were conducted using the released variant call format call set. Variants in the FD gene (*GLA*, located on the X chromosome) were extracted from the TMM GRCh38 call set and annotated using ClinVar (release 2023-02-26).^10^ Variant pathogenicity was classified according to ClinVar clinical significance, with variants labeled as Pathogenic or Likely pathogenic considered pathogenic and those labeled as Benign or Likely benign considered nonpathogenic. Variants classified as Uncertain significance or Conflicting interpretations were excluded from the primary pathogenic definition and evaluated separately where relevant. Because *GLA* is located on the X chromosome, genotype interpretation accounted for sex, with males treated as hemizygous and females as heterozygous or homozygous as applicable.

## Results

Among 68,615 individuals (mean age, 47.7 ± 19.5 years; 66% female), 4 participants with pathogenic *GLA* variants (mean age, 49.3 ± 15.9 years) and one participant aged 70 to 79 years with a likely pathogenic variant were identified, corresponding to a prevalence of 1 in 13,723; all were female (Table 1). All pathogenic variants were c.902G>A, whereas the likely pathogenic variant was c.1076T>C. Among the 5 heterozygotes, renal function data were available for 4, with a mean estimated glomerular filtration rate of 102.3 ± 21.2 mL/min/1.73 m^2^. Microalbuminuria was observed in 1 participant (case 3). Electrocardiographic data were available for 3 heterozygotes; no left ventricular hypertrophy was identified based on standard voltage criteria (RV5 ≥ 26 mm or RV5 + SV1 ≥ 35 mm). Heterozygotes with pathogenic *GLA* variants had a mean plasma GLA activity of 5.64 ± 1.73 mmol/h/mL, whereas the heterozygote with the likely pathogenic variant had a plasma GLA activity of 8.36 mmol/h/mL. Plasma globotriaosylsphingosine (lyso-Gb3) levels were 3.7 ± 0.79 ng/mL in those with pathogenic variants and 0.96 ng/mL in the individual with the likely pathogenic variant (Table 1). The identified variants of uncertain significance (*n* = 17; 1 in 4,036) included p.Ala15Thr (*n* = 8), p.Asp153Asn (*n* = 6), p.Gln212Arg (*n* = 2), and p.Asn419Asp (*n* = 1). No variants with conflicting interpretations were identified in this analysis.

**Table 1:** Clinical characteristics of 5 participants with Fabry disease–causing variants

Case	Age (years)	Sex	dbSNP ID	Variant	Protein Change	Disease Type	Serum GLA Activity (mmol/h/mL)	Serum Lyso Gb3 (ng/mL)	LVH	eGFR (mL/min/1.73m^2^)	Urinary Albumin (mg/gCr)
1	30s	Female	rs104894828	c.902G>A	p.Arg301Gln	Late-onset	5.64	2.67^a^	-	NA	NA
2	40s	Female	rs104894828	c.902G>A	p.Arg301Gln	Late-onset	6.92	4.60^a^	-	132.9	22.2
3	50s	Female	rs104894828	c.902G>A	p.Arg301Gln	Late-onset	3.20	3.82^a^	NA	114.2	48.6
4	70s	Female	rs104894828	c.902G>A	p.Arg301Gln	Late-onset	6.80	3.71^a^	NA	88.1	15.1
5	70s	Female	rs1928132914	c.1076T>C	p.Ile359Thr	likely-pathogenic	8.36	0.96	-	74.1	6.8

LVH was defined according to electrocardiographic voltage criteria as RV5 + SV1 ≥ 35 mm or RV5 ≥ 26 mm.

*dbSNP*, single nucleotide polymorphism database; *GLA*, α-galactosidase A; *lyso-Gb3*, globotriaosylsphingosine; *LVH*, left ventricular hypertrophy; *eGFR*, estimated glomerular filtration rate; *NA*, not available.

a Exceeded the upper limit of normal (normal upper limit ≤2.0 ng/mL).

## Discussion

In this study, genome sequencing in a large Japanese community cohort from the TMM Project identified pathogenic or likely pathogenic *GLA* variants in 1 of every 13,723 individuals, indicating a higher prevalence of FD–associated variants than was historically estimated from clinically ascertained populations.^1^ All identified heterozygotes were female, and all pathogenic variants were c.902G>A,^11^ suggesting that late-onset FD, particularly among women, may remain underrecognized in the general population. Historically, the prevalence of FD has been estimated at 1 in 40,000 to 1 in 170,000 based on clinical diagnosis,^1^ whereas population-based genomic data from the UK Biobank showed a substantially higher prevalence of 1 in 5573.^12^ Although the prevalence observed in our cohort was lower than that reported in the UK Biobank, this difference may reflect ethnic variation in *GLA* variant distribution and differences in cohort composition between European and Japanese population-based studies. Nevertheless, both studies consistently suggest that FD–causing variants are more common than was previously recognized.^3^ Newborn screening studies similarly report prevalences ranging from approximately 1 in 1600 to 1 in 11,854, largely driven by late-onset variants.^4^^,^^13^, ^14^, ^15^, ^16^ Together, these findings support the presence of a substantial reservoir of undiagnosed FD in the general population.

Plasma lyso-Gb3, a disease-related metabolite and potential biomarker of therapeutic response, exceeded the upper limit of normal (≤2.0 ng/mL)^17^ in all 4 participants with c.902G>A, supporting biochemical relevance despite the absence of overt clinical manifestations. We previously reported marked clinical heterogeneity among patients with c.902G>A in the same region^2^ and its repeated detection in the present cohort may reflect regional enrichment of this late-onset phenotype.

No hemizygous males with pathogenic *GLA* variants were identified. One possible explanation is that males with clinically overt disease may have already been diagnosed and therefore were less likely to participate in this community-based cohort. We also identified 1 likely pathogenic variant (case 5, c.1076T>C), previously reported in FD but with conflicting evidence regarding its pathogenicity.^18^^,^^19^ In our cohort, the heterozygote showed normal lyso-Gb3 levels, no left ventricular hypertrophy, and preserved renal function without microalbuminuria, suggesting a limited phenotypic impact of this variant. These findings support the value of longitudinal follow-up in population-based biobanks to clarify the clinical penetrance of FD–associated variants, particularly in women with late-onset genotypes. Information on family history and cascade screening of relatives was not available in the present biobank-based analysis. Therefore, we could not determine how many family members were found to be at risk or newly diagnosed through the identification of these probands. Future linkage with clinical follow-up and family-based evaluations may help clarify the broader diagnostic impact of genotype-first screening.

In conclusion, population-based genome sequencing in the TMM revealed that pathogenic or likely pathogenic FD variants are more prevalent than previously recognized, indicating that late-onset FD in women may remain substantially underdiagnosed.

## Declaration of Generative AI and AI-Assisted Technologies in the Writing Process

During the preparation of this work, the author (KN) used ChatGPT (OpenAI) for English language editing. After using this tool/service, the authors reviewed and edited the content as needed and take full responsibility for the content of the publication.
